# Survival of Ectoparasitic Mites *Tropilaelaps mercedesae* in Association with Honeybee Hive Products

**DOI:** 10.3390/insects10020036

**Published:** 2019-01-29

**Authors:** Kitiphong Khongphinitbunjong, Panuwan Chantawannakul, Orlando Yañez, Peter Neumann

**Affiliations:** 1 School of Science, Mae Fah Luang University, Chiang Rai 571000, Thailand; kitiphong.kho@mfu.ac.th; 2 Department of Biology, Faculty of Science, Chiang Mai University, Chiang Mai 50200, Thailand; panuwan@gmail.com; 3 Institute of Bee Health, Vetsuisse Faculty, University of Bern and Agroscope, Schwarzenburgstrasse 161, CH-3097 Bern, Switzerland; orlando.yanez@vetsuisse.unibe.ch

**Keywords:** *Tropilaelaps mercedesae*, comb, pollen, trade

## Abstract

The global trade of honeybee hive products imposes the risk of the introduction of exotic pests. However, data on the potential of specific products enabling pest survival are often lacking. This holds especially true for ectoparasitic mites *Tropilaelaps* spp., which are mandatory pests of honeybees in many countries. Here, we evaluated the longevity of *Tropilaelaps mercedesae* mites associated with empty honeycomb and dry pollen as two possible global import routes. Mites were able to survive up to three days in dry pollen and up to six days in empty honeycomb, thereby suggesting a sufficient time window for the potential introduction of *T. mercedesae* into mite-free countries via import of these hive products.

## 1. Introduction

The international trade of the honeybee hive products honey, pollen, royal jelly, propolis, bee venom, and beeswax can play a significant role in the spread of diseases or infectious agents [1], thereby creating demand for adequate action by the local and international authorities. *Tropilaelaps* spp. are ectoparasitic mites of honeybees *Apis* spp., endemic to Asia, which have the potential to develop into a global threat to wild and managed honeybees [2]. Even though the trade of beekeeping tools, package bees, queen cages, and bee colonies are the most likely route for the introduction of *Tropilaelaps* spp. into new areas [1], the import of bee products may also pose a risk. The World Organisation for Animal Health (OIE) currently recommends restricting the trade and handling of honeybee products infested by *Tropilaelaps* spp. mites [3]. Chapter 9.5, “Infestation of honeybees with *Tropilaelaps* spp.,” regulates the importation of several commodities including beeswax and bee-collected pollen. It is therefore mandatory to present an international veterinary certificate attesting that the commodities come from apiaries situated in a country or zone free from *Tropilaelaps* mites or that appropriate measures were taken to ensure the destruction of the mites. However, the importation by travelers of small amounts of products that may be untreated or treated with non-appropriated methods against mites almost certainly represents a high risk. Heat processed beeswax may be less of a concern for the introduction of some pests (but see small hive beetles, [4]), but unprocessed honeycomb may represent a higher risk. Although the importation of honeycomb containing bee brood is forbidden in many countries, honeycomb without bee brood may be allowed to enter a country in a terminal pass-check.

Since adult *Tropilaelaps* spp. mites can only feed on immature bees [5], their survival can vary significantly if transported on adult honeybee workers (up to three days, [6]), pupae (up to five days, [7]) or larvae (up to four weeks, [8]). However, information is currently limited about survival of these mites associated with honeybee products. Therefore, we tested survival of *Tropilaelaps* mites associated with honeycomb and dry pollen in the absence of honeybees.

## 2. Materials and Methods

The study was conducted in Chiang Mai (Thailand) in June and July 2017. DNA analysis confirmed the experimental mites to be *Tropilaelaps mercedesae* [9]. Adult female mites (N = 200) were sampled from a single, heavily-infested *Apis mellifera* colony by uncapping freshly sealed worker brood [10] pooled in a petri dish and kept with freshly obtained 5th instar honeybee larvae prior to individual transfer to the honeybee products. Pieces of dark honeycombs (6 × 13 cm) were prepared from empty honey frames, which were kept at −4 °C and kept dry prior to usage. Both honeycomb and dry pollen packages (N = 5 per honeybee product) received 20 female *T. mercedesae* by carefully introducing them individually with fine brushes. Then, the pieces of honeycomb were completely wrapped with plastic film and placed individually in plastic bags (Ziploc^®^, JOHNSON & SON, Racine, Wisconsin, USA) to simulate trading packages. Dry pollen (25 g) was filled in plastic containers (80 cm^3^), which were covered with a lid. Dry pollen was used instead of fresh pollen because this represents the international trading standard to limit the spread of diseases [1]. Then, all packages were randomly placed in an incubator (to limit possible spatial effects) and kept at 25 °C and 70% relative humidity. All packages were inspected daily by opening and subsequently sealing the plastic film cover again and dead mites removed until all mites have died.

All statistical analyses were performed using the NCSS 10 statistical software (2015, NCSS, LLC, East Kaysville, UT, USA). Since the data were not normally distributed (Kolmogorov-Smirnov Test, *p* = 0.268), non-parametric tests were used for differences in mite survivorship. Treatments were compared using log Rank multiple test and visualized with Kaplan Meier curves.

## 3. Results

**Figure 1:**
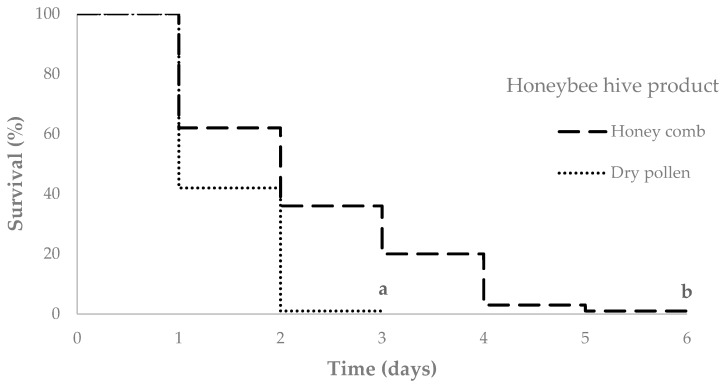
Kaplan-Meier curve displaying the survival of *Tropilaelaps mercedesae* mites associated with honeycomb and dry pollen in the absence of honeybees. Significant differences are indicated by different letters (a,b, Log Rank test, χ2= 33.237, df = 1, *p* < 0.0001).

The last mites died after six days associated with honeycomb and three days with pollen packages, respectively (Figure 1). No significant differences were found within replicates for either honeycomb (2.22 ± 0.27 days, Log Rank test, *χ^2^*= 7.281, df = 4, *p* = 0.1218), or dry pollen (1.43 ± 0.11 days, Log Rank test, *χ^2^*= 3.587, df = 4, *p* = 0.4647). Therefore, all five replications were integrated for the survival analyses. The mites survived significantly longer on honeycomb (median = 2 and interquartile range = 2) compared to dry pollen (median = 1 and interquartile range = 1, Log Rank test, *χ^2^*= 33.237, df = 1, *p* < 0.0001).

## 4. Discussion

The data clearly show that *T. mercedesae* mites were able to survive up to three days in dry pollen and up to six days in honeycomb, thereby suggesting that international transportation might enable the global spread of this mandatory pest.

*T. mercedesae* survival rates differed significantly between the products. Honeycomb proved more suitable for mite survival, enabling a twice as long survival compared to dry pollen. Previous studies have shown that *T. clareae* cannot survive for more than 48 h without feeding on honeybee brood [11], which is similar to our average survivorship of 34 h on dry pollen. However, a few mites were found alive above the average after introduction to honeycomb. The results strongly suggest that mite survival can be extended depending on the honeybee product. Thus, it seems plausible that survivorship of *T. mercedesae* is not only dependent on food from the bees, but also on temperature and relative humidity of the environment, i.e. hosting products (the container with dry pollen may provide lower humidity). Since we used 70% relative humidity, this may have promoted mite survival and probably represents a worst-case scenario. 

Irrespective of the traded product, the *T. mercedesae* maximal survival suggests a sufficient time window for global spread via the fastest mode of transport, i.e. air freight (about two days). This risk should be taken into consideration to limit the global spread of this mandatory pest, either via sufficient quarantine (six days minimum, but further evaluation seems required to play safe) in exporting countries and/or adequate treatment of traded hive products (see [3]).

## 5. Conclusions

Our data clearly show that *T. mercedesae* mites were able to survive up to three days in association with dry pollen and up to six days associated with empty honeycomb, thereby suggesting a sufficient time window for the potential introduction into mite-free countries via import of these hive products. Adequate border control for these products is therefore suggested.
